# Integrin Activation: Implications for Axon Regeneration

**DOI:** 10.3390/cells7030020

**Published:** 2018-03-10

**Authors:** Menghon Cheah, Melissa R. Andrews

**Affiliations:** 1 John van Geest Centre for Brain Repair, University of Cambridge, Cambridge CB2 0PY, UK; mc747@cam.ac.uk; 2 Centre for Developmental Neurobiology, King’s College London, London SE1 1UL, UK; 3 Department of Biological Sciences, University of Southampton, Life Sciences Bldg 85, Highfield Campus, Southampton SO17 1BJ, UK

**Keywords:** extracellular matrix, gene therapy, integrin activation, kindlin, regeneration, talin, tenascin

## Abstract

Integrin activation is essential for creating functional transmembrane receptors capable of inducing downstream cellular effects such as cell migration, cell spreading, neurite outgrowth and axon regeneration. Integrins are bidirectional signalling molecules that mediate their effects by ‘inside–out’ and ‘outside–in’ signalling. This review will provide a detailed overview of integrin activation focusing on intracellular activation in neurons and discussing direct implications in the regulation of neurite outgrowth and axon regeneration.

## 1. Introduction

Integrins are transmembrane receptors that form cell–cell or cell–matrix interactions in order to instigate vital cellular events such as cell migration, cell spreading and, more recently, neurite outgrowth and axon regeneration. Integrin receptor binding and subsequent receptor activation are critical for these processes to occur. Both the developing and adult nervous systems are affected by integrin-mediated interactions. For example, during cortical development, integrins are required for neuronal migration during the process of laminar organisation and synaptogenesis [1,2]. In astrocytes, integrins are required for cell adhesion, differentiation and migration during nervous system development and maintenance of the mature nervous system [3]. Myelinating cells such as oligodendrocytes and Schwann cells also express integrins which are directly involved in coordinating the process of axon myelination [4,5].

As integrins are essential for the proper functioning of a normal and healthy nervous system, translational researchers in the field of axon regeneration have been trying to harvest the use of integrins following a central nervous system (CNS) injury, such as spinal cord injury, in order to recapitulate a developmental growth state that could enhance regenerative growth. In the past decade, we have published a series of progressive studies that demonstrate the potential use of integrins for promoting both neurite outgrowth and in vivo axon regeneration in the spinal cord following injury [6,7,8]. Included in these data is the pivotal finding that although forced expression of integrin subunits alone can promote substantial regeneration, the activation state of the integrin receptor needs to be considered in order to fully maximise regenerative potential. In the event of CNS injury, the upregulation of inhibitory molecules such as chondroitin sulfate proteoglycans (CSPGs), myelin debris and Nogo-A in the lesioned environment can result in integrin inactivation, hence relinquishing any axon-promoting effects [9,10,11]. In this review, we will discuss how an integrin receptor functions, with emphasis on its activation state and the implications that integrin activation has on promoting axon regeneration. 

## 2. The Challenging Task of Axon Regeneration 

The adult mammalian CNS is well known for its inability to regenerate after injury due to the limiting factors found in both the nervous system environment and within the neurons themselves. This is in contrast to the peripheral nervous system (PNS) and the early developing CNS [12,13,14] where axon regeneration and plasticity are not only possible but are also likely to occur. In order to achieve functional axon regeneration after injury, processes such as growth cone formation, response to guidance cues in the environment, synaptogenesis and axon pruning are required. These processes are similar to those of developing axons for which integrins play a large part. However, there are spatiotemporal regulations throughout CNS maturation that dictate the expression of particular integrin subunits or heterodimers [15]. As a result, some integrin subunits are downregulated after nervous system development, thereby having knock-on effects on the ability of adult neurons to form certain cell–matrix interactions and thus inhibiting neurite outgrowth and axon regeneration required for regrowth. The involvement of integrins in neuronal development, maturation and post-injury expression has been reviewed in detail elsewhere [16]. 

The diverse integrin family allows for the binding of a plethora of ligands in the extracellular matrix (ECM) and has, therefore, made integrin receptors ideal candidates for neurite outgrowth and axon regeneration in terms of flexibility and specificity. As a family, integrins can recognise more than 20 different ligands in the ECM as well as on neighbouring cells [17]. During in vivo neurite outgrowth or axon regeneration, the growing axon may have to grow through a long list of ECM ligands on its path to the terminal target. Thus, optimising the expression and activation of a transmembrane receptor such as an integrin that can recognise so many different ligands would be very useful for nervous system repair. 

The neurons of the dorsal root ganglion (DRG) have been a popular model for researchers studying integrin and neurite outgrowth. The unique characteristic of DRG neurons with axonal projections both in the periphery (peripheral branch) and the spinal cord (central branch) has made them a useful model for studying the underlying mechanisms for successful axon regeneration in the PNS environment and also the failure of axon regeneration in the adult mammalian CNS. Successful PNS regeneration of DRG neurons after injury has been demonstrated with an upregulation of specific integrin subunits such as α4, α5, α6, α7 and β1 [18,19,20,21]. Furthermore, it has been shown that embryonic/developing DRG neurons can modulate their integrin expression in response to different concentrations of a ligand, such as laminin [22], as well as adapt their integrin expression in the presence of an inhibitory substrate such as CSPG in order to achieve maximum neurite outgrowth [23]. The fact that embryonic DRG neurons possess this dynamic regulation of integrin expression and subsequent outgrowth has prompted the integrin manipulation in adult DRG neurons to promote adult CNS axon regeneration [24]. Firstly, to further understand how integrin activation can have an implication on axon regeneration, it is essential to understand how integrin receptors function as signalling molecules. 

## 3. Integrin Heterodimers as a Transmembrane Signalling Molecule 

A functional integrin receptor is a heterodimer made up of an alpha (α) and a beta (β) subunit. The integrin family comprises 18 α and 8 β subunits that are non-covalently associated into 24 different heterodimers [25]. Different integrin heterodimers bind to different ligands such as laminin, collagen, fibrinogen, tenascin-C and cadherin [17,26]. Most integrin heterodimers can recognise more than one ligand and the same ligand can bind to different heterodimers. 

**Figure 1:**
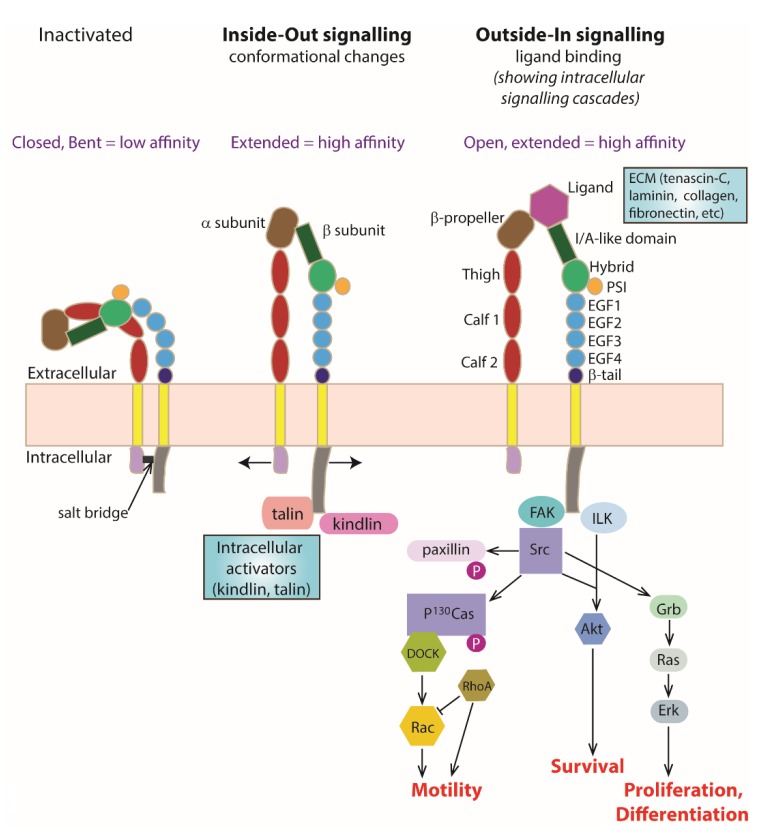
**Integrin Structure and Activation.** Activation of integrin heterodimers leads to intracellular signalling cascades and resulting processes such as cell motility, cell survival, cell differentiation, cell differentiation and neurite outgrowth. Schematic representing integrin conformations at the membrane including changes that occur with ‘Inside–Out signalling’ and ‘Outside–In signalling’. An inactivated integrin heterodimer exists with a closed and bent conformation (extracellularly) stabilised by a cytoplasmic salt bridge. This conformation has a very low ligand binding affinity. With Inside–Out signalling, intracellular activators (such as kindlin and talin) bind the β subunit cytoplasmically and interact/destabilise the salt bridge, leading to an open and extended (active) conformation with increased ligand binding affinity. With Outside–In signalling, binding of a ligand (ECM molecules such as laminin, fibronectin, or tenascin) extracellularly occurs as a result of integrin activation leading to a conformational change to an open and extended (active) conformation with high ligand binding affinity. Individual names of the extracellular domain components have been shown in the Outside–In signalling example for simplicity, with further explanation in the main text.

Each integrin subunit contains a large extracellular domain, a transmembrane domain and a cytoplasmic domain. Briefly, the extracellular domain of the α subunit consists of a β-propeller, thigh, calf-1 and calf-2 domains [27]. In addition, a subset of α subunits such as α1, α2, α10 and α11 also has an I/A domain within the β-propeller. The I/A domain contains a metal ion-dependent adhesion site (MIDAS) that acts as a ligand binding site [28,29]. Metal ions such as the cations magnesium, calcium or manganese can be found binding in the MIDAS. On the other hand, the extracellular domain of the β subunit consists of an I/A domain, an immunoglobulin-like hybrid domain, a plexin-semaphorin-integrin (PSI) domain, four EGF-like repeats (EGF1-4) and a β-tail domain [27]. For those α subunits that do not have an I/A domain, the I/A domain located on the β subunit serves as the ligand binding site instead, after forming a complex with the β-propeller on the α subunit [30] (Figure 1).

Integrins are bidirectional signalling molecules. They exist in either the inactivated or activated state. It has been suggested that integrins are transported to the plasma membrane from the endoplasmic reticulum as a heterodimer in the bent inactivated state [31]. During integrin activation, the molecule changes from a bent low-affinity conformation into a stable extended high-affinity conformation, known as ‘inside–out’ signalling. This results in a higher affinity binding of extracellular ligands to the activated integrin and triggers a series of intracellular signalling cascades that are important for relaying information from the external environment to the inside of the cell, termed as ‘outside–in’ signalling (Figure 1).

The integrin cytoplasmic domains, especially the β subunit cytoplasmic domain, are important in regulating the affinity state of the receptor and triggering intracellular signalling cascades. When the receptor is in the bent inactivated low-affinity state, a salt bridge is formed between the KLLITIHD motif on the β subunit and the GFFKR motif on the α subunit [32]. Binding of an activator to the β subunit cytoplasmic domain unclasps these two cytoplasmic domains and induces affinity and conformational changes in the receptor [33] (Figure 1).

## 4. ‘Inside–Out’ Signalling

The process that induces a conformational change in the integrin receptor from a bent inactivated low-affinity state to a stable extended activated high-affinity state via an intracellular activator is termed ‘inside–out’ signalling or integrin activation. Upon integrin activation, the ligand binding site on the I/A domain, which is normally hidden in the inactivated state, is exposed to the surrounding environment for ligand binding [33]. The two well-known intracellular activators of integrin are kindlin and talin. Both kindlin and talin contain a FERM (4.1/ezrin/radixin/moesin) domain that has a phosphotyrosine binding (PTB) site serving as the integrin binding site [34,35].

### 4.1. Kindlin

Three members have been identified in the kindlin family: kindlin-1, kindlin-2 and kindlin-3. These isoforms differ in terms of subcellular localisation and expression [36]. For instance, kindlin-1 is expressed predominantly in epithelial cells, kindlin-2 is ubiquitously expressed and is the only kindlin isoform present in the nervous system, and kindlin-3 is expressed almost exclusively in haematopoietic cells such as leukocytes and macrophages. The FERM domain is located at the C-terminus of kindlin and is made up of F1, F2 and F3 subdomains [37]. All kindlin isoforms bind to the membrane-distal NxxY motif on the β subunit cytoplasmic tail of integrin via the F3 subdomain at the PTB site, resulting in a conformational change and activation of the integrin receptor. Functionally, kindlin was first observed to colocalise with integrin and play a role in integrin-dependent cell–matrix adhesion in *C. elegans* [38].

Most studies on kindlin have focused on its role in epithelial cells and leukocytes, with very few studies being performed on the nervous system. We have recently published two related studies that have clearly shown the growth-promoting role of kindlin in the nervous system as it relates to integrin expression and activation [7,8]. Specifically, overexpression of kindlin-1 in adult rat DRG neurons promotes sensory axon regeneration by overcoming the growth-inhibiting effect of the CSPG, aggrecan. This was achieved by increasing the level of integrin activation demonstrated with increased levels of phosphorylated FAK (by immunostaining with anti-pY397). Previously, CSPGs have been shown to have a direct effect on integrin inactivation, although the underlying cellular mechanism is not clear [11]. Interestingly, overexpression of kindlin-2, the isoform usually present in the nervous system, did not result in any axon growth [7]. This suggests that the kindlin isoforms presumably have different and non-redundant functions from each other, despite their structural similarities.

### 4.2. Talin

In vertebrates, there are two forms of talin: talin-1 and talin-2. Talin-1 is expressed in all cell types with talin-2 being found primarily in the brain, skeletal and cardiac muscles [39]. Talin is a 270 kD protein composed of a head domain (≈50 kD) and a tail domain (≈220 kD). On the talin head, there is a FERM domain that binds to the membrane-proximal NPxY motif on the β subunit integrin cytoplasmic tail. This binding separates the cytoplasmic tails of the α and β subunits, leading to a conformational change of the integrin receptor and integrin activation [40]. This is similar to the ‘inside–out’ signalling of kindlin. However, unlike talin, kindlin does not possess a tail domain. The talin tail domain can self-regulate its integrin-binding site on the head domain [41]. In addition, the tail domain contains binding sites for F-actin and vinculin, which are components of the cytoskeleton at focal adhesions. Talin has been suggested as a scaffolding protein that holds multiple integrin-associated proteins and the cytoskeleton together at the focal adhesions as a large complex, triggering a series of intracellular events and cell motility [42]. Hence, talin is widely considered to be a more potent integrin activator than kindlin due to the additional cellular functions associated with the talin tail domain. A recent study has demonstrated that kindlin alone is unable to unclasp the cytoplasmic tails of α and β subunits for integrin activation, unlike talin [43]. It is also possible that talin and kindlin may have distinct roles in regulating the function of integrins [44]. 

Despite its higher potency in activating integrin, many studies have chosen to study talin by focusing on either the head or tail domain [43,45,46]. This is very likely due to the large size of the entire molecule (≈2500 amino acids), which makes experimental procedures such as cell transfection of full-length talin difficult. However, to utilise the full function of talin in activating integrins to promote neurite outgrowth, the full-length talin molecule is required. In its full length, talin can achieve integrin activation and stimulate neurite outgrowth on inhibitory CSPG substrates when overexpressed in cultures of adult rat DRG neurons [47]. Since this study has only been confirmed in cell culture, its potential for in vivo axon regeneration is yet to be investigated. 

### 4.3. Intracellular Interactions with Kindlin and Talin

Although it is clear that kindlin binds to integrin on the membrane-distal NxxY motif of the β subunit cytoplasmic tail and talin binds to the membrane-proximal NPxY motif, the interactions between integrin, kindlin and talin are not yet well understood. It is possible that kindlin and talin work independently or as co-activators [48,49]. Three models have so far been proposed for the synergistic activation of integrins by talin and kindlin [50]:

(1) The sequential binding model: Kindlin binds to the membrane-distal NxxY motif to induce a slight change in the conformation of the β subunit cytoplasmic tail and this facilitates the binding of talin to the membrane-proximal NPxY motif.
(2) The *Cis* co-operation model: Simultaneous binding of kindlin and talin to the same β subunit integrin cytoplasmic tail via their respective binding sites.
(3) The *Trans* co-operation model: Kindlin and talin each bind to different β subunit cytoplasmic tails and then interact with each other to form integrin clustering at focal adhesions.

## 5. ‘Outside–In’ Signalling

**Figure 2:**
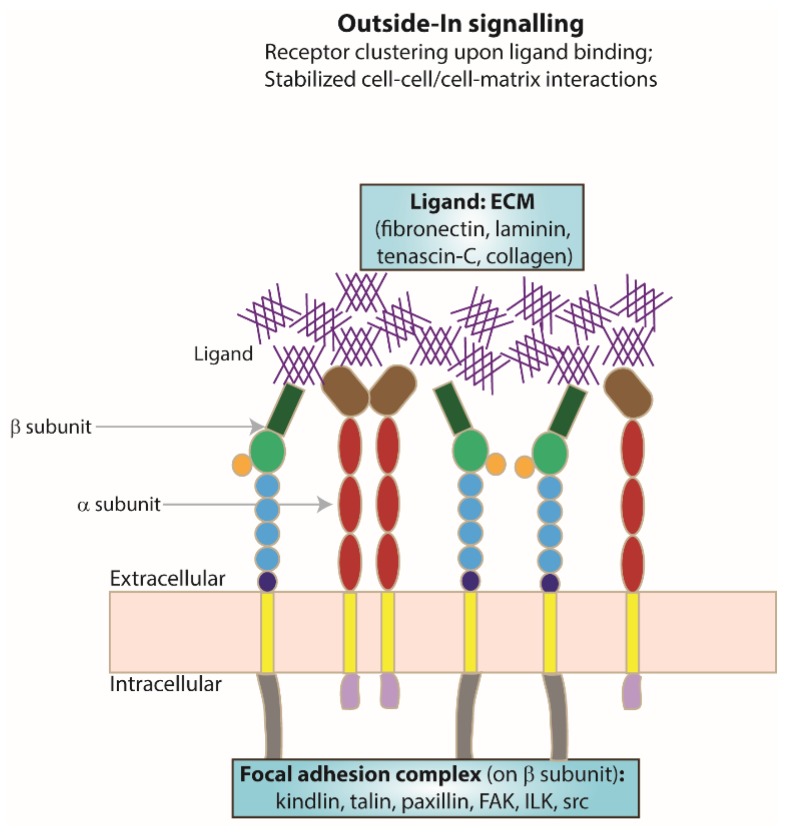
**Focal adhesion formation.** Schematic representing integrin receptor clustering at the membrane as an example of receptor activation, a result of ligand (ECM) binding and one of the resulting changes following ’outside–in’ signalling.

As a result of integrin activation, high-affinity binding of an extracellular ligand to the activated integrin receptor can occur. Upon ligand binding, integrin clustering occurs as part of the focal adhesion required to stabilise cell–cell or cell–matrix interactions (Figure 2). The formation of focal adhesions triggers a series of intracellular signalling cascades for responses ranging from short-term effects, such as cell adhesion and motility, to long-term effects, such as cell proliferation and differentiation, which may include changes in gene expression. As integrin cytoplasmic tails do not possess any kinase or phosphatase activities, the transduction of intracellular signalling cascades is mediated by molecules such as focal adhesion kinase (FAK) [51] and the adaptor protein integrin-linked kinase (ILK) [52]. 

### Extracellular Ligand Binding

Integrin receptors comprise a huge and complex family of proteins. Part of this complexity is derived by, as previously mentioned, the fact that different integrin heterodimers bind to different ligands with most heterodimers able to recognise more than one ligand and also the same ligand can be recognised by different integrin heterodimers (Reviewed by [17,26]). From our previous studies on integrins and axon regeneration, we focus mainly on the heterodimer α9β1 [6,8]. The α9β1 integrin binds to osteopontin and tenascin-C (TN-C) to mediate cell–matrix interactions, as well as VCAM-1 for cell–cell interactions. In addition, osteopontin is also recognised by α4β1, α5β1, αVβ1 and αVβ5, while TN-C is also recognised by α8β1 and αVβ3, as well as others. The list of integrin ligands continues to grow as more integrin–ligand interactions are being discovered.

The presence or absence of a particular integrin heterodimer on the cell directly affects its ability to grow on a particular ECM substrate. It was observed that motor and sensory neurons have different preferences for substrates at early postnatal stages due to the difference in integrin heterodimer expression [53]. For instance, sensory neurons tend to extend longer neurites on laminin matrices due to the expression of the laminin receptor α7β1 integrin, while motor neurons were observed to grow better on fibronectin matrices due to the expression of the fibronectin receptor α5β1 integrin. However, this difference was lost in the adult [53]. This finding has highlighted the fact that different ligands can have different integrin-mediated growth effects on the neurons, and the expression of integrin is subject to both temporal and spatial regulations. 

## 6. Other Factors That Can (Artificially) Modulate Integrin Function

### 6.1. Divalent Cations

Divalent cations have various effects on ligand affinity of integrin receptors ranging from enhancement to suppression. The cation calcium (Ca^2+^) mainly functions intracellularly and induces an inhibitory effect on ligand binding. Specifically, Ca^2+^ has been shown to stabilise the closed and bent conformation of the integrin heterodimer, creating low or null binding affinity of these receptors for the ECM and other ligands [54,55]. On the other hand, certain divalent cations such as manganese (Mn^2+^) and magnesium (Mg^2+^) have been shown to modulate integrin activity by their interactions and binding to the extracellular domain. Complete extension of the extracellular domain and subsequent separation of the cytoplasmic domains lead to intracellular signalling events such as induction of cell adhesion, migration and neurite outgrowth [24,56]. Ligand binding leads to an increase in ligand affinity as well as an increase in receptor clustering and recruitment at the membrane, which may further contribute to increased ligand affinity, leading to the use of Mn^2+^ to artificially activate integrins in cell culture assays. For example, Mn^2+^ binding to integrin was shown to induce a 2–10 fold increase in integrin receptor affinity and specificity for fibronectin [57].

In cell culture, activation of endogenous and/or overexpressed integrins enhances neurite outgrowth significantly more than the expression of integrins alone in the presence of inhibitory substrates [7,8,11,58]. Specifically, the application of Mn^2+^ to adult dorsal root ganglia (DRG) cultures grown in the presence of the inhibitory proteoglycan aggrecan led to significantly increased neurite outgrowth compared to untreated cultures [11,58]. 

### 6.2. Integrin-Activating Antibodies

There are a whole host of integrin antibodies available commercially. Depending on the recognition site and/or resultant conformational change following the binding of these antibodies, some of these antibodies have an inhibitory effect on integrin function, some have a stimulatory or activation effect on integrin function, and some purely recognise a specific epitope on an integrin subunit. The latter are well suited for biochemical and immune-based investigation of integrin expression, whereas the others can be utilised to modulate integrin activity and are commonly monoclonal. Although it is outside the scope of this review, inhibitory antibodies act mainly as competitive inhibitors for ligand binding, thus blocking or significantly reducing integrin interactions with ECM and other ligands (Reviewed by [59]). It is because of this functionality that monoclonal integrin blocking antibodies are being used therapeutically in various cardiac conditions and cancer, to name a few. 

In terms of integrin activation using specific monoclonal antibodies, the majority recognises the β subunit (with a small minority having α subunit specificity) with many of these inducing a conformational change to the integrin heterodimer that results in opening up or exposure of a ligand binding site. Activation can be further dictated by cation or ligand binding in a portion of these antibodies such as the HUTS-4 antibody for β1 integrin, whereas some are cation/ligand-independent such as the TS2/16 antibody, also for β1 integrin. As these antibodies function at the extracellular domain of the integrin, in terms of the β subunit, this would involve the PSI domain and the EGF-like repeats, all of which are obscured in the bent conformation (Reviewed by [59]). Therapeutically, less work has been done in pushing integrin-activating antibodies to the clinic although there is strong evidence to show that the use of these antibodies (specifically the human-specific antibody, TS2/16) to artificially induce activation can significantly enhance neurite outgrowth of cultured human embryonic stem cell-derived motor neurons grown on inhibitory proteoglycan-rich substrates over untreated cultures [11].

## 7. Our Findings on Integrin Activation and Axon Regeneration

Our initial experiments on integrin and axon regeneration were based on previous studies observing ECM glycoproteins such as CSPGs and TN-C upregulated at the lesion site after spinal cord injury. Recognising that these molecules pose both a biochemical and physical barrier for axon regeneration, we began our research into overcoming the barrier presented by these molecules to achieve functional axon regeneration. In doing so, we have studied the effect of both integrin expression and integrin activation in the context of nervous system injury and repair, examining axon regeneration as well as behavioural and functional recovery. 

The α9 integrin subunit is a receptor for TN-C. In the CNS, α9 integrin is downregulated upon maturation. In the injured CNS however, TN-C is upregulated without the subsequent expression of the α9 integrin subunit [6] contributing to a detrimental imbalance and failed CNS regeneration. Using adult rat dissociated DRG sensory neurons as a model, transgenic expression of α9 integrin resulted in these neurons growing vigorously on a TN-C substrate, similar to control cultures grown on growth-permissive laminin [6]. When the same hypothesis was tested in vivo using adult rat dorsal root or dorsal column crush injury and examining sensory axon regeneration, α9 integrin-expressing DRG neurons regenerated into the TN-C-rich dorsal root entry zone (DREZ) or TN-C-rich lesion site, respectively, with no axonal growth observed beyond these areas [6]. The different levels of neurite outgrowth observed in cell culture (significant) versus the axon regeneration observed in vivo (modest but above control levels) raised the question as to whether the overexpressed α9 integrin had been deactivated in the injured CNS. 

After CNS injury, growth inhibitory molecules such as CSPGs and Nogo-A are highly upregulated at the lesion site. As mentioned earlier, both of these lesion site factors have been shown to inactivate integrins [7,10,11]. Specifically, we and others examined integrin activation in relation to the inhibitory CSPG, aggrecan, and/or Nogo-A using adult rat DRG neurons [7,11] or motor neurons derived from human embryonic stem cells [11] and showed that both can directly affect integrin activation to effectively reduce neurite outgrowth. In these studies, the levels of phosphorylated FAK (pY397) were reduced in cells cultured in the presence of aggrecan or Nogo-A with the level of total FAK remaining unchanged, suggesting a significant reduction in integrin activation [7,11]. Fortunately, this can be overcome by experimentally inducing integrin activation via the application of Mn^2+^ or an integrin-activating antibody [11] or via transgenic expression of an integrin intracellular activator, kindlin-1 [7]. As both of these studies did not investigate integrin directly, it was left to speculation whether the overexpressed α9 integrin in the previous study of in vivo axon regeneration had been deactivated and maintained in the inactivated state due to CSPGs and Nogo-A at the lesion site, suggesting a rationale for the modest regeneration observed.

After demonstrating that both α9 integrin and kindlin-1 can promote the regrowth of injured axons, we next examined whether combined expression of α9 integrin and the activator kindlin-1 could promote long-distance functional axon regeneration further than the expression of either alone [8]. In dissociated adult rat DRG cultures expressing α9 integrin only, there was a modest amount of neurite outgrowth observed when plated on a mixed substrate of both CSPG (aggrecan) and TN-C [8]. In contrast, when DRG cultures expressed both α9 integrin and kindlin-1, the resultant neurite outgrowth on the substrate of CSPG and TN-C was significantly increased. This result suggests that the overexpressed α9 integrin was indeed deactivated in the injured CNS in our initial α9 integrin study. Subsequently, we examined the effect of co-expression of α9 integrin and kindlin-1 in vivo using adult rat lower cervical dorsal root crush injury (C5-C8) as our model. When adeno-associated viruses (serotype 5; AAV5) expressing α9 integrin and kindlin-1 were co-injected directly into the DRGs concurrent with the dorsal root injury, we observed long-distance axon regeneration of DRG sensory axons up to 25 mm into the spinal cord by 12 weeks post-injury [8]. The axons grew beyond the TN-C-rich region and, in addition, they also formed topographically accurate connections in the dorsal horn of the spinal cord and continued to grow rostrally within the dorsal columns of the spinal cord. The time point at which we examined the combined treatment group was 12 weeks post-injury, 6 weeks longer than the time points tested in the previously published studies examining regeneration with α9 integrin alone or kindlin-1 alone. Despite this, either α9 integrin alone or kindlin-1 alone were tested alongside the combined treatment group, also with a 12-week post-operative timeline. No additional regeneration was observed in the single treatment groups above the regeneration observed in the original 6-week studies. Regeneration of the major subtypes of sensory neurons was observed including large-diameter NF200- (neurofilament 200 kD) positive neurons and small-diameter CGRP- (calcitonin gene-related peptide) and isolectin IB4-positive neurons. In addition, functional recovery and thus regenerative growth was evident following electrophysiological analysis. Furthermore, neuroanatomical analysis demonstrated significant regenerative growth into the caudate nucleus in the caudal medulla, while behavioural recovery was demonstrated through tasks such as ladder-walking (for limb control and coordination), von Frey mechanical pressure and Hargreaves thermal tasks (for mechanical and pain sensation, respectively) [8].

In summarising our findings (Table 1), the presence of an appropriate integrin heterodimer on the neuronal surface determines whether a regenerating neuron possesses the ability to grow on a particular ECM molecule. However, it should also be stressed that the activation state of the integrin receptor should be taken into consideration in order to induce maximal integrin-dependent neurite outgrowth and/or axonal regeneration.

## 8. The Outlook for Nervous System Repair

We have demonstrated that modulating the expression and activation of integrins in neurons presents a promising therapeutic approach to promoting neurite outgrowth and axon regeneration [6,7,8]. Moving forward clinically, one may question the feasibility of translating our findings into a clinical treatment. Although the use of gene therapy in the clinic is still in its infancy, there may be the potential for integrin-mediated treatment to be introduced into viable therapies alongside other growth-promoting strategies for spinal cord repair. 

It is clear that the activation state of integrin receptors is an important factor in attaining significant regenerative growth, especially and in particular in the presence of inhibitory molecules. We demonstrated this with the expression of the intracellular integrin activator, kindlin-1 [7,11]; however, integrin activation can also be achieved by using integrin-activating antibodies. These antibodies, as mentioned above, act to enhance integrin signalling extracellularly by inducing a conformational change in the receptor and thereby increasing ligand affinity/binding and promoting neurite outgrowth. The majority of integrin-activating antibodies are human specific, which, although does not work for experimental rodent models, already work for human cells. Further proof-of-concept experiments utilising human stem cell cultures as a model will continue to move the field forward towards translation. Several antibodies (human and humanised) that induce inhibition of specific factors/proteins are already in use clinically. Examples include natalizumab against α4β1 integrin in immune-mediated conditions such as multiple sclerosis and Crohn’s disease [60,61], and alemtuzumab against the lymphocyte antigen CD52, also for multiple sclerosis [62]; while antibodies in clinical trials include anti-Nogo A against myelin protein Nogo-A for the treatment of spinal cord injury [63].

Furthermore, although it has not been touched on in this review, consideration for the localisation and transport of overexpressed integrin receptors within the axonal compartment of neurons is also vital for maximising integrin-mediated regenerative potential [64,65]. Nonetheless, the recapitulation of a developmental growth state in the CNS to enhance regeneration following traumatic injury is potentially possible through modulation of appropriate integrin expression and activation within damaged neurons.

## Figures and Tables

**Table 1:** Summary of integrin-mediated dorsal column axon regeneration.

**Publication**	Andrews et al., 2009	Tan et al., 2012	Cheah et al., 2016
**Molecule**	α9 integrin	Kindlin-1	α9 integrin + kindlin-1
**In vitro model**	Adult rat dissociated dorsal root ganglia (DRG) neurons plated on laminin (control) or tenascin-C (TN-C).	Adult rat dissociated DRG neurons plated on laminin (control) or aggrecan.	Adult rat dissociated DRG neurons plated on laminin (control), aggrecan, TN-C, or aggrecan + TN-C.
**In vitro results**	Neurite outgrowth when grown on TN-C rescued by expression of α9 integrin to levels similar to growth on laminin. Growth was significantly higher than wildtype neurons grown on TN-C.	Neurite outgrowth when grown on aggrecan rescued by expression of kindlin-1 to levels similar to growth on laminin. Growth was significantly higher than wildtype neurons grown on aggrecan.	Neurite outgrowth of DRG neurons when grown on aggrecan + TN-C rescued by combined expression of α9 integrin and kindlin-1 to levels similar to growth on laminin. Growth was significantly higher than neurons expressing α9 integrin or kindlin-1 alone grown on aggrecan + TN-C.
**In vivo model**	Unilateral cervical dorsal root crush injury (C5–C8) *, examined 6 weeks post-injury.	Unilateral cervical dorsal root crush injury (C5–C8), examined 6 weeks post-injury.	Unilateral cervical dorsal root crush injury (C5–C8); examined 12 weeks post-injury **.
**Virus transduction**	AAV2-α9 integrin injected into C6, C7 DRGs. AAV2-fGFP as control.	AAV2-kindlin1-mCherry injected into C6, C7 DRGs. AAV2-mCherry and AAV2-fGFP as controls.	AAV5-kindlin1-GFP and AAV-α9integrin-V5 injected into C6, C7 DRGs. AAV5-fGFP as control.
**Anatomical results**	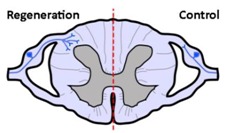	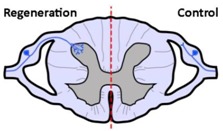	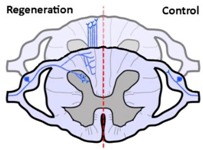
	α9 integrin-expressing axons grew into the TN-C-rich DREZ. Control axons did not grow into the CNS at all.	Kindlin1-expressing axons grew beyond the DREZ and into the dorsal horn. Control axons did not grow into the CNS at all.	Axons co-expressing α9 integrin and kindlin-1 grew beyond the TN-C-and-CSPG-rich DREZ and into the dorsal horn topographically, and also within the spinal cord (cuneate fasciculus) to the medulla for a distance of up to 25 mm. Control axons did not grow into the CNS at all.
**Behavioural tests**	Behavioural recovery to pre-operative levels **in α9 integrin group** in thermal pain sensory test.	Behavioural recovery to pre-operative levels **in kindlin-1 group** occurred in both the mechanical pressure and thermal pain sensory tests.	Significant behavioural recovery to near pre-operative levels **in combined treatment group** in mechanical pressure and thermal pain sensory tests, and ladder-walking (limb proprioception) test.
**Electro-physiology**	N/A	N/A	Significant functional reconnection shown between injured dorsal roots and associated dorsal horn **in combined treatment group**.

* Cervical (C4–C5) dorsal column crush lesions also performed in a separate group of experiments. ** Cheah et al. also performed experiments with α9 integrin alone and kindlin1 alone for direct comparison to the combined treatment group for a 12-week duration, but no additional regeneration was observed over the results observed in Andrews et al. and Tan et al.
